# Supplementary material for: Antibiotic dispensing behaviors for pediatric diarrhea: A qualitative study of informal healthcare providers in rural Bangladesh
Source: PLoS One. 2025 Dec 18;20(12):e0339283. doi: 10.1371/journal.pone.0339283 (PMC12714273; doi:10.1371/journal.pone.0339283)
Supplement: Attachment — Submitted filename: Reviewers attachement.docx [file pone.0339283.s001.docx]

**Introduction:**

1. Please write the practice of antibiotic use for treating diarrhoea by physicians and informal providers globally
2. Please provide clinical explanation of antibiotic use in pediatric diarrhoea treatment
3. Please include misuse and overuse of antibiotics in Bangladesh
4. Please write the practice of antibiotic use for treating diarrhoea by physicians in Bangladesh
5. Please expand the last paragraph that includes the necessity of exploring antibiotic use in pediatric diarrhoea treatment by informal providers, and necessity of qualitative exploration (with proper citation)

**Method:**

6. “Sitakunda was selected as a study site because of our team’s familiarity with the area and established working relationships with members of the community”-this should not be the reasons of conducting study in a particular area. Please explain other reasons that convince us to choose this area.
7. “This study is part of a larger initiative focused on developing and evaluating a mobile health tool to improve antibiotic stewardship among village doctors in Bangladesh- please mention this at the begging of the method and explain the relevancy of the study with the larger initiative.
8. We need to know the context of the study areas such as healthcare facilities, accessibility of the healthcare facilities, literacy of the people, economic status, education, profession etc
9. Please explain why you selected only 20 village doctors for IDI among large population (371 village doctors). Why you did not use other qualitative tools such as FGD, observations and case study.Why did not you include patients and their parents and drug sellers in the study?

**Result:**

10. You mentioned 20 village doctors in the methods, but now you mention 18. Please make clear in the manuscript.
11. Demographic information is not sufficient. You need to include education, training or source of knowledge, income from the treatment etc.
12. In Bangladesh, village doctors provide treatment and provide antibiotic for both human and animal. Do you have any findings related to that?
13. It is necessary to know the knowledge and perception of negative impact of using antibiotic and antibiotic resistance from village doctors. Please write details about that with quotation.
14. Do the village doctors have any conflict with formal healthcare providers? Please explain.
