# Supplementary material for: Antibiotic dispensing behaviors for pediatric diarrhea: A qualitative study of informal healthcare providers in rural Bangladesh
Source: PLoS One. 2025 Dec 18;20(12):e0339283. doi: 10.1371/journal.pone.0339283 (PMC12714273; doi:10.1371/journal.pone.0339283)
Supplement: Attachment — Submitted filename: Response letter to reviewers_R1.docx [file pone.0339283.s003.docx]

**Response to Reviewers**

Manuscript ID PONE-D-25-34609

We would like to thank the reviewers for their time in reviewing our manuscript “**Antibiotic dispensing behaviors for pediatric diarrhea: A qualitative study of informal healthcare providers in rural Bangladesh**”. Our team appreciates the thoughtful reviews and are grateful for the opportunity to revise and resubmit our manuscript.

We have carefully reviewed the comments and feedback provided by each reviewer and have revised the manuscript accordingly. Our revisions are made using tracked changes as instructed. We have also submitted a clean version of the revised paper.

We look forward to the next review of this manuscript and the opportunity to have it published in *PLOS ONE*.

**Academic Editor**

1. Please ensure that your manuscript meets PLOS ONE's style requirements, including those for file naming.

**Response**: We have thoroughly reviewed the formatting and made necessary changes in the revised manuscript.

2. Thank you for stating the following financial disclosure: “Funding for this study was provided through a grant from the Eunice Kennedy Shriver National Institute of Child Health and Human Development of the US National Institutes of Health (1R21HD109819 to DTL, EJN, and AIK).”Please state what role the funders took in the study.  If the funders had no role, please state: "The funders had no role in study design, data collection and analysis, decision to publish, or preparation of the manuscript."If this statement is not correct you must amend it as needed. Please include this amended Role of Funder statement in your cover letter; we will change the online submission form on your behalf.

**Response:** We have edited the financial disclosure to reflect the role that the funders took in the study. The revised statement is shown below

“Funding for this study was provided through a grant from the Eunice Kennedy Shriver National Institute of Child Health and Human Development of the US National Institutes of Health (1R21HD109819 to DTL, EJN, and AIK). The funders had no role in study design, data collection and analysis, decision to publish, or preparation of the manuscript.”

3. We note that you have indicated that there are restrictions to data sharing for this study. PLOS only allows data to be available upon request if there are legal or ethical restrictions on sharing data publicly. For more information on unacceptable data access restrictions, please see http://journals.plos.org/plosone/s/data-availability#loc-unacceptable-data-access-restrictions.Before we proceed with your manuscript, please address the following prompts:

**a)** If there are ethical or legal restrictions on sharing a de-identified data set, please explain them in detail (e.g., data contain potentially identifying or sensitive patient information, data are owned by a third-party organization, etc.) and who has imposed them (e.g., a Research Ethics Committee or Institutional Review Board, etc.). Please also provide contact information for a data access committee, ethics committee, or other institutional body to which data requests may be sent.

**b)** If there are no restrictions, please upload the minimal anonymized data set necessary to replicate your study findings to a stable, public repository and provide us with the relevant URLs, DOIs, or accession numbers. For a list of recommended repositories, please see

https://journals.plos.org/plosone/s/recommended-repositories. You also have the option of uploading the data as Supporting Information files, but we would recommend depositing data directly to a data repository if possible.

We will update your Data Availability statement on your behalf to reflect the information you provide.

**Response:** Although our data does not contain directly identifying information, we have chosen not to release the data in a public repository given the nature of qualitative data. Uploading our qualitative data would require submitting interview transcripts that include personal narratives, including details about employment history and place of practice. Someone who is familiar with this setting/context could use this information to identify the study participants. We have updated our data availability statement to include how to obtain the qualitative data if desired.

“**Data Availability Statement:** The dataset is under the custodianship of the Research Administration at icddr,b. A de-identified analytical data set will be made available upon requests directed to the Research Administration of the icddr,b, at [info@icddrb.org](mailto:info@icddrb.org). Only after approval of a proposal data can be shared through a secure online platform. Approval of the proposal will be subject to scientific review by the institutional review board at icddr,b. Sharing of data will also be subject to the published data access rules of the icddr,b. The requestor will need to sign a standard data access agreement required by the icddr,b.”

4. Please note that funding information should not appear in any section or other areas of your manuscript. We will only publish funding information present in the Funding Statement section of the online submission form. Please remove any funding-related text from the manuscript.

**Response**: We have removed funding information from the manuscript texts.

5. Please include your full ethics statement in the ‘Methods’ section of your manuscript file. In your statement, please include the full name of the IRB or ethics committee who approved or waived your study, as well as whether or not you obtained informed written or verbal consent. If consent was waived for your study, please include this information in your statement as well.

**Response:** We have included the full names of the IRBs from both institutions and noted that written consent was obtained from participants before conducting interviews.

**“**The study protocol was approved by the Ethical Review Committee of the International Centre for Diarrhoeal Diseases Research, Bangladesh (icddr,b), and the Institutional Review Board of the University of Utah, USA. The investigators explained the study to each participant and obtained informed written consent before conducting the interviews.” (Lines 239-243)

6. If the reviewer comments include a recommendation to cite specific previously published works, please review and evaluate these publications to determine whether they are relevant and should be cited. There is no requirement to cite these works unless the editor has indicated otherwise.

**Response:** Not applicable

7. Please review your reference list to ensure that it is complete and correct. If you have cited papers that have been retracted, please include the rationale for doing so in the manuscript text, or remove these references and replace them with relevant current references. Any changes to the reference list should be mentioned in the rebuttal letter that accompanies your revised manuscript. If you need to cite a retracted article, indicate the article’s retracted status in the References list and also include a citation and full reference for the retraction notice.

**Response:** We have checked the reference lists carefully.

**Reviewer 1:**

***Introduction***
1. Please write the practice of antibiotic use for treating diarrhoea by physicians and informal providers globally

**Response:** We appreciate this suggestion. We agree that providing a broader perspective on global practices of antibiotic use for diarrhea enhances the contextual understanding of antibiotic misuse, particularly in LMICs. We have revised the introduction to include a more detailed discussion of how physicians and informal providers manage diarrhea with antibiotics globally with relevant citations.

“Globally, both formally trained physicians and informal providers frequently prescribe antibiotics for non-bloody, non-severe diarrhea, driven by diagnostic uncertainty, perceived caregiver expectations and limited time for counselling [9-13]. Studies from South and Southeast Asia, sub-Saharan Africa, and Latin America consistently report high rates of antibiotic recommendation or dispensing for uncomplicated pediatric diarrhea in both formal and informal sectors, with substantial between-country variation but a shared pattern of overuse [14-17].” (Lines 102-108)

2.Please provide clinical explanation of antibiotic use in pediatric diarrhoea treatment

**Response**: We appreciate the reviewer’s suggestion and have expanded our explanation to include clinical rationale for antibiotic use in pediatric diarrhea.

“Currently, decisions for using antibiotics and laboratory testing for acute diarrhea in children are mostly empiric. Although the World Health Organization (WHO) Integrated Management of Childhood Illness (IMCI) guidelines recommend antibiotics only for cases of bloody diarrhea or suspected cholera [9], inappropriate antibiotic use remains widespread. (Lines 97-101)

3.Please include misuse and overuse of antibiotics in Bangladesh

**Response**: Thank you for this important suggestion. We have addressed this by including a more detailed description of antibiotic misuse and overuse in Bangladesh, supported by relevant references.

“In Bangladesh, the misuse and overuse of antibiotics are common, particularly through informal providers and over-the-counter sales without prescriptions [10, 18-20]. This practice often involves broad-spectrum antibiotics for minor, viral illnesses, driven by caregiver demand and pharmaceutical marketing [10, 21-23].” (Lines 111-115)

4.Please write the practice of antibiotic use for treating diarrhoea by physicians in Bangladesh

**Response:** While this study focuses on informal healthcare providers, evidence from Bangladesh indicates that inappropriate antibiotic prescribing for pediatric acute diarrhea also occurs within formal facilities, despite guideline recommendations emphasizing ORS and zinc. In addition to our response to the above comment (Reviewer 1, comment 3), we have included a statement describing antibiotic practices within the formal healthcare system of Bangladesh.

“Moreover, evidence from Bangladesh indicates that inappropriate antibiotic prescribing for acute pediatric diarrhea also occurs within formal facilities, despite guideline recommendations emphasizing that in most cases treatment should be limited to ORS and zinc supplementation [24,25].” (Lines 115-118)

5.Please expand the last paragraph that includes the necessity of exploring antibiotic use in pediatric diarrhoea treatment by informal providers, and necessity of qualitative exploration (with proper citation)

**Response**: Thank you for this valuable suggestion. We have expanded the final paragraph of the introduction to emphasize the importance of investigating antibiotic use in pediatric diarrhea treatment by informal providers, and the critical role of qualitative research in this context.

“Understanding antibiotic use in pediatric diarrhea treatment by informal providers is essential, particularly in settings where they serve as the primary source of care. Since informal providers often operate outside of formal regulatory frameworks, their antibiotic prescribing behaviors can be shaped by various factors. Qualitative methods such as in-depth interviews, focus group discussions and ethnographic observation are well suited for understanding how social factors contribute to the inappropriate use of antibiotics and barriers to guideline adherence [5, 29]. These insights are crucial for designing context-specific antibiotic stewardship programs, strategies, and interventions. (Lines 132-140)

Method:

6.“Sitakunda was selected as a study site because of our team’s familiarity with the area and established working relationships with members of the community”-this should not be the reasons of conducting study in a particular area. Please explain other reasons that convince us to choose this area.

**Response**: Thank you for this important feedback. We have revised the paragraph to provide a more comprehensive rationale for selecting the study site.

“The study was conducted in Sitakunda Upazila, a sub-district of Chattogram District in the southeastern part of Bangladesh. Sitakunda Upazila is approximately 500 square kilometers of land area and has a population of around 383,000 [32]. Sitakunda was selected as a study site because it has high pediatric diarrhea burden [32]. The area has a large number of informal healthcare providers in practice in areas with a broad range of health care access and economic conditions. Our team knows the area well due to decades of public health programming in Chattogram, and has built strong, established working relationships with members of the community through previous research.” (Lines 162-173)

7.“This study is part of a larger initiative focused on developing and evaluating a mobile health tool to improve antibiotic stewardship among village doctors in Bangladesh- please mention this at the beginning of the method and explain the relevancy of the study with the larger initiative.

**Response**: Thank you for this helpful suggestion. We have revised the text. We have now explained this at the beginning of our methods section.

“This study is part of a larger project aimed at creating and testing a mobile health (mHealth) tool that supports village doctors in Bangladesh to use antibiotics responsibly [30]. The broader initiative includes mapping village doctors, conducting formative qualitative research to understand providers’ behavior, co-designing the tool with users, pilot testing, and evaluating its impact. As the formative research phase, the current study utilized a qualitative descriptive design to examine…” (Lines 148-154)

8. We need to know the context of the study areas such as healthcare facilities, accessibility of the healthcare facilities, literacy of the people, economic status, education, profession etc

**Response:** We appreciate this thoughtful comment. In response to the suggestion, we have added a description of the study areas in the manuscript.

“The study was conducted in Sitakunda Upazila, located in the Chattogram District of southeastern Bangladesh. Sitakunda covers an area of around 500 km² with a population of approximately 457,396 [32]. The literacy average rate in Sitakunda Upazila was about 54%. The primary occupations of the local population are in the service sector (28.76%), agriculture (24.12%), and commerce (21.53%). The hospitals and healthcare centers in Sitakunda Upazila include Union Health centre and community clinics at the wards [32].” (Lines 162–168)

9.Please explain why you selected only 20 village doctors for IDI among large population (371 village doctors). Why you did not use other qualitative tools such as FGD, observations and case study. Why did not you include patients and their parents and drug sellers in the study?

**Response**: We appreciate this thoughtful comment. Our team has previously conducted work in this region and published a study (citation 34) that identified 411 village doctor sites, from which 371 village doctors agreed to complete structured surveys. Please refer to our responses below regarding sample size, data collection techniques, and inclusion of other stakeholders.

*Regarding sample size*:  In qualitative research, adequacy is based on richness and saturation rather than population size; prior methodological work suggests 10–17 IDIs within a relatively homogeneous group are typically sufficient to reach saturation and identify major patterns. We monitored saturation iteratively and observed no new themes emerging after approximately 15 interviews, with three additional interviews confirming the stability of findings. We have added these details to the methods.

“We targeted recruiting 20 village doctors because prior methodological work suggests 10-17 in-depth interviews within a relatively homogeneous group are typically sufficient to reach data saturation and identify major themes. [35,36]” (Lines 184-187)

*Regarding data collection techniques***:** The decision to use in-depth interviews instead of other qualitative approaches, such as focus group discussions (FGDs), was driven by the goals of our research. IDIs allowed for a deeper exploration of individual perspectives and personal experiences relating to antibiotic prescribing practices. Given the sensitive nature of the inquiry (i.e., potentially inappropriate antibiotic use), individual interviews enabled participants to express their views without peer influence. FGDs could have constrained individual reflections due to group dynamics, possibly leading to conformity rather than candid discussion. Observations and case studies were not employed as they would necessitate extensive time and resources not feasible within the project's scope, especially given the immediate need to address antibiotic resistance in the community context. We have added the following text to the methods section:

“We used in-depth interviews rather than other qualitative methods like focus group discussions due to the specific aim and context of our study. Individual interviews allow us to dive deeper into each participant’s thoughts and experiences about antibiotic prescribing practices. This was especially important given the sensitivity of the possible misuse of antibiotics. By discussing one-on-one, participants could share their opinions freely, without the pressure of a group setting [37, 38].” (Lines 195-201)

*Regarding inclusion of other stakeholders***:** Our research focused specifically on the perspectives and practices of village doctors since they are directly responsible for dispensing antibiotics in rural settings. The aim was to comprehensively understand how their knowledge, beliefs, and external pressures shape their practices, which forms a critical link in addressing antibiotic resistance. Future studies would benefit from a more inclusive approach that incorporates feedback from these stakeholders, facilitating a holistic understanding of the antibiotic supply chain.

“Moreover, we interviewed village doctors only as our research focused specifically on the perspectives and practices of village doctors since they are a first point of care in rural settings.” (Lines 187-189)

Results:

10.You mentioned 20 village doctors in the methods, but now you mention 18. Please make clear in the manuscript.

**Response**: Thank you for this comment. We acknowledge that this is a confusing point in our manuscript. We contacted 20 village doctors; 18 agreed to participate and completed in-depth interviews, while two declined due to time constraints. This information is included in the method section of our revised manuscript.

“From the 20 village doctors who were approached, 18 agreed to participate in the study and completed in-depth interviews. The remaining two declined due to time constraints” (Lines 190-191)

11.Demographic information is not sufficient. You need to include education, training or source of knowledge, income from the treatment etc.

**Response**: We have not reported this information here because the detailed demographic and practice characteristics of village doctors in this study—including education, training background, sources of medical knowledge, income from treatment, and practice patterns—have been reported in our previous publication. We have added a brief statement in the result section of our revised manuscript with citation (citation 34).

12.In Bangladesh, village doctors provide treatment and provide antibiotic for both human and animal. Do you have any findings related to that?

**Response**: We did not collect data on village doctors' provision of antibiotics for animal use in this study. Our interview guide and analysis were focused specifically on antibiotic dispensing for pediatric diarrhea in humans, aligned with the objectives of the broader mHealth intervention targeting human health care. Due to this, we have not made any changes to the manuscript.

13.It is necessary to know the knowledge and perception of negative impact of using antibiotic and antibiotic resistance from village doctors. Please write details about that with quotation.

**Response**: We appreciate the reviewer's interest in village doctors' knowledge and perceptions of antibiotic resistance. This finding emerges under the individual level of the Social Ecological Framework, where we present findings that highlight knowledge gaps and misconceptions about antibiotics, supported by direct quotations from participants. Notably, many participants had a limited understanding of what constitutes an antibiotic; for example, some did not recognize metronidazole as an antibiotic. Additionally, there were misconceptions about the spectrum of antibiotics, with some believing that antibiotics are effective against all pathogens, including viruses. We did not create a separate standalone section on "knowledge and perceptions of resistance" because knowledge emerged as one aspect of the broader individual-level determinants in our ecological framework, alongside attitudes and perceived benefits.

14.Do the village doctors have any conflict with formal healthcare providers? Please explain.

**Response**: Although some participants briefly mentioned referral practices or interactions with formal healthcare facilities, we did not gather enough data to describe the nature, extent, or impact of any conflicts or collaborative relationships that village doctors have with formal healthcare providers. We recognize this as a limitation and have added a point in the Discussion section suggesting that future research should explore the dynamics among informal and formal providers and how these dynamics may influence antibiotic stewardship. We added the following text:

“This study did not examine how village doctors and formal healthcare providers interact or if there are any conflicts. Issues such as competition, referrals, respect and collaboration can create both barriers and facilitators to appropriate antibiotic use. Future research should explore these relationships to help create better strategies for addressing antibiotic resistance.” (Lines 557-561)

**Reviewer #2:**

Thanks for developing a manuscript titled "Antibiotic dispensing behaviors for pediatric diarrhea: A qualitative study of informal healthcare providers in rural Bangladesh", which is crucial for the sector. Overall, the paper is well written. However, please find some specific areas where we have the opportunity to improve:

1. Line 82: Are they village doctors? or medicine dispensers? What is the qualification of these village doctors? Please add this somewhere at the beginning of the paper. Also, you may clarify for the reader that village doctors and shop dispenser are different.

**Response**: Thank you for this thoughtful question. We have added more description in the revised manuscript to clarify the term “village doctors” in the context of Bangladesh.

“In the context of rural Bangladesh, “village doctors” are informal healthcare providers who play a central role in primary care, particularly in underserved areas. They often operate small, private clinics or medicine shops where they both recommend and directly provide medications, including antibiotics, to patients. Village doctors have no formal medical qualifications, often learning through apprenticeship, short training courses, or self-study. They differ from drug shop dispensers, who sell medications without providing clinical consultation or diagnosis.” (Lines 77-84)

2. Line 84: Private clinic and medicine shops are not same, so please edit "or", maybe "and" is appropriate here

**Response**: Thank you for noticing this. We have modified the sentence, which now reads:

“They often operate small, private clinics and/or medicine shops where they both recommend and directly provide medications, including antibiotics, to patients.” (Lines 79-80)

3. Line 86: Important target for whom? Government? The entire sector? One health?

**Response**: We have modified the statement in the revised manuscript which now reads:

“This group is an important target of antibiotic stewardship efforts by public health authorities, antimicrobial resistance (AMR) control programs, and the broader health sector in Bangladesh and similar low- and middle-income countries [2,3].” (Lines 84-92)

4. Line 111: Community health worker introduced here for the first time, may be better to add one sentence on them earlier to this when you mention village doctors. i. e. Who are these community health workers? Why are they more qualified than village doctors?

**Response**: We appreciate this comment and have modified the sentences in the revised manuscripts to address this.

“…compared to 30% of those who consulted a community health worker (government-supported lay health workers with standardized training who provide basic health services and referrals but do not independently dispense medications) “ (Lines 126-127)

5. Lines 117-119: Maybe it's better to explain the research gap here first, and then describe how qualitative research can contribute.

**Response**: We appreciate this comment. As this suggestion is similar to a previous comment (Reviewer 1, comment 5), please see our response and revisions there.

6. 137: It seems that this is not a stronger justification for selecting the field site. Maybe the next paragraph is talking about the justification of the field sites, which seems good.

**Response**: We have revised the section to include a more detailed description of how we select field sites. As this suggestion is similar to a previous comment (Reviewer 1, comment 6), please see our response and revisions there.

7. Figure: The quality of the figure should be better; it's distorted now.

**Response**: We have updated our figure with a higher resolution to enhance clarity.
