# Supplementary material for: Antibiotic dispensing behaviors for pediatric diarrhea: A qualitative study of informal healthcare providers in rural Bangladesh
Source: PLoS One. 2025 Dec 18;20(12):e0339283. doi: 10.1371/journal.pone.0339283 (PMC12714273; doi:10.1371/journal.pone.0339283)
Supplement: Attachment — Submitted filename: Response_letter_to_reviewers_R1_auresp_2.docx [file pone.0339283.s004.docx]

**Response to Reviewers/Editor**

Manuscript ID PONE-D-25-34609R1

Dear Editor,

Thank you for the opportunity to revise and resubmit our manuscript.

As advised in your recent correspondence, we understand that no new reviewer or editor comments were provided for this round of review. We confirm that in our previous revision, we have carefully addressed all reviewer comments and editorial concerns raised in the earlier round of peer review.

Accordingly, we have not made any further changes to the manuscript at this stage.

We remain grateful for the constructive feedback received during the review process and hope that the revised manuscript will now be suitable for publication in PLOS ONE.

Thank you for your continued consideration.

Sincerely,

Debashish Biswas
